# Severe Pertussis During Early Infancy from a High-Altitude Region: Two Clinical Cases and Literature Review

**DOI:** 10.3390/jcm15062211

**Published:** 2026-03-14

**Authors:** Hongju Chen, Sezhen Baima, Xiaoming Xu, Tao Wang, Jing Shi

**Affiliations:** 1 Department of Pediatrics, West China Second University Hospital, Sichuan University, Chengdu 610041, China; cilla_8597@163.com (H.C.); xuxiaoming19920617@163.com (X.X.); 2 Xizang Region Child Development Clinical Medical Research Center, Xizang Hospital of West China Second University Hospital, Sichuan University & Xizang Autonomous Region Women’s and Children’s Hospital, Lhasa 851400, China; 18217796121@163.com; 3 Key Laboratory of Birth Defects and Related Diseases of Women and Children (Sichuan University), Ministry of Education, West China Second University Hospital, Sichuan University, Chengdu 610041, China; 4 Department of Pediatrics, Xizang Hospital of West China Second University Hospital, Sichuan University & Xizang Autonomous Region Women’s and Children’s Hospital, Lhasa 851400, China

**Keywords:** pertussis, infant, high altitude, hyperleukocytosis, pulmonary arterial hypertension, exchange transfusion

## Abstract

**Objective**: To investigate how the high-altitude environment modifies severe pertussis in young infants and analyze its pathophysiological mechanisms and clinical management implications. **Methods**: Clinical data of two young infants with severe pertussis residing at 3650 m were retrospectively analyzed, including presentation, laboratory findings, pathogen detection, treatment, and outcomes. A literature review explored synergistic interactions between high-altitude factors and pertussis pathophysiology. **Results**: Case 1 had macrolide-resistant *Bordetella pertussis* (MRBP, 23S rRNA A2047G) with peak WBC 52.25 × 10^9^/L, and received cefoperazone-sulbactam, piperacillin-tazobactam and azithromycin, and was successfully treated with trimethoprim-sulfamethoxazole combined with exchange transfusion. Case 2 had *Bordetella pertussis* confirmed by PCR with peak WBC 36.55 × 10^9^/L, receiving cefoperazone-sulbactam and azithromycin, and recovered. Both developed respiratory failure requiring non-invasive ventilation and survived without pulmonary hypertension. High-altitude stressors—hypoxia, enhanced pulmonary vascular reactivity, and hypercoagulability—synergize with pertussis-induced hyperleukocytosis as a “dual hit,” accelerating cardiopulmonary deterioration and elevating thrombotic risks. **Conclusions**: High altitude is an independent risk modifier in infantile pertussis, demanding heightened vigilance and proactive interventions: early non-invasive ventilation, prophylactic anticoagulation, and timely exchange transfusion before pulmonary hypertension develops. This is the first high-altitude case series that provides essential insights for clinicians in similar environments globally, guiding early recognition and proactive management strategies to improve outcomes in this vulnerable population.

## 1. Introduction

Pertussis is a respiratory infectious disease caused by *Bordetella pertussis* (*B. pertussis*) that affects individuals of all ages. Newborns and infants under three months, however, face the highest risk of severe complications due to their immature immune systems and incomplete vaccination schedules [1]. The classic clinical course of pertussis comprises catarrhal, paroxysmal, and convalescent stages, with major complications including pneumonia, hypoxia, encephalopathy, and apnea. Severe pertussis is characterized by hyperleukocytosis, refractory hypoxemia, pulmonary arterial hypertension (PAH), and cardiogenic shock, all contributing to a poor prognosis [2]. Immunologically immature neonates bear the greatest global pertussis disease burden, with a mortality rate of up to 3% [3]. Data from the United States also indicate a pertussis case-fatality rate of 1–2% in neonates and infants under 2 months of age [4].

Current research on pertussis is largely based on populations in low-altitude regions. Management experience for high-risk groups under extreme conditions, such as those residing at high altitude, remains limited, particularly for newborns and young infants. This vulnerability may be further amplified in high-altitude settings. The hypobaric and hypoxic conditions at high altitude can adversely affect pulmonary function [5]; consequently, in infants with pertussis, this hypoxia compromises compensatory mechanisms during paroxysmal coughing. Additionally, dry and cold air impairs mucociliary clearance and secretion expulsion, creating a vicious cycle that exacerbates hypoxia. Furthermore, recurrent hypoxemia induced by pertussis can precipitate severe PAH, and the ambient hypoxic environment at high altitude may further accelerate the development of post-infectious PAH, thereby increasing right ventricular afterload and predisposing patients to heart failure. The onset of pertussis in young infants is often atypical, and disease progression can be especially rapid in high-altitude settings, leading to severe illness that requires prompt recognition and intervention.

The primary aim of this report is to highlight how high-altitude conditions uniquely influence the clinical presentation, progression, and management of severe pertussis in young infants, with a particular focus on the compounded risks of hyperleukocytosis, PAH, and thrombotic complications in this vulnerable population. Here, we present the first documented case series of two young infants with pertussis from a high-altitude region. One case featured rapidly progressive hyperleukocytosis, while the other was a neonate with macrolide-resistant pertussis and hyperleukocytosis who was successfully treated (Table 1). This series provides valuable insights for the diagnosis and clinical management of pertussis under such extreme environmental conditions.

## 2. Case Presentations

### 2.1. Case 1

**Figure 1:**
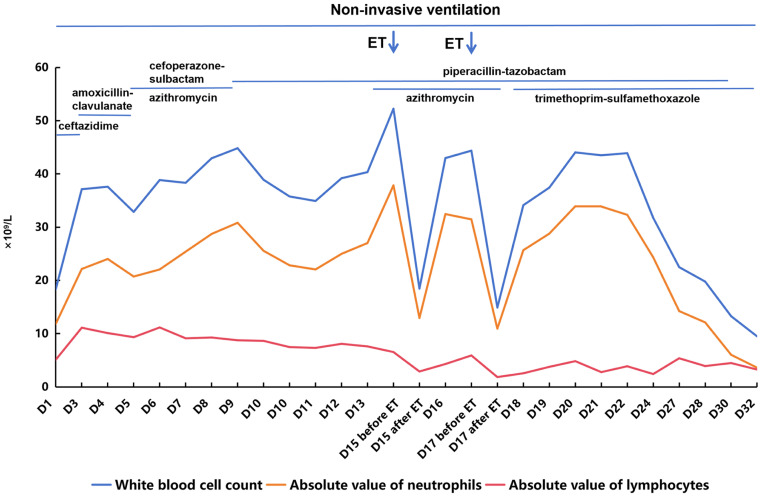
Changes in blood routine parameters and treatment of Case 1. ET: exchange transfusion.

**Figure 2:**
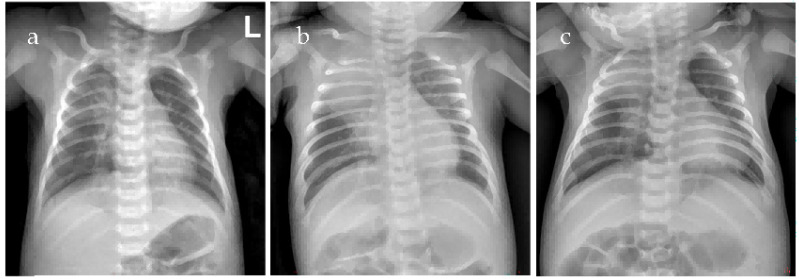
Chest radiograph images of Case 1 (**a**–**c**). Image (**a**) obtained at admission demonstrates mild patchy opacity, while both (**b**) obtained prior to the first bronchoscopy and (**c**) obtained prior to the second bronchoscopy reveal bilateral faint patchy opacities and consolidation, predominantly in the right upper lung zone.

A 12-day-old female neonate was admitted in winter 2024 due to poor responsiveness for 2 days and cough for 1 day. Born at 37 weeks and 5 days of gestation with a birth weight of 2940 g, she was the second child (Gravida 3, Para 2, G3P2). Delivered vaginally with unremarkable amniotic fluid, placenta, umbilical cord, and Apgar scores. Parents are healthy; maternal pregnancy was uncomplicated. The mother had received a full childhood course of diphtheria, tetanus, acellular pertussis (DTaP) vaccination. An older sibling had a recent upper respiratory tract infection. Initial presentation included a single harsh cough, inspiratory retractions, auscultation revealed moist rales in the right lung, tachycardia (heart rate (HR) 182 beats per minute (bpm)), tachypnea (respiratory rate (RR) 71 breaths per minute (rpm)), cyanosis on room air, and fever (38.2 °C). Laboratory tests revealed a white blood cell (WBC) count of 18.35 × 10^9^/L with an absolute neutrophil count (ANC) of 11.84 × 10^9^/L (64.5%) and lymphocytes of 5.11 × 10^9^/L (27.8%), along with C-reactive protein (CRP) of 23.84 mg/L (Figure 1), procalcitonin (PCT) of 6.804 ng/mL, and hyponatremia. Chest radiograph revealed bilateral consolidation (Figure 2). A diagnosis of severe pneumonia was made, and she was started on ceftazidime and non-invasive ventilation (NIV). Sputum culture grew *Haemophilus* influenzae, prompting a switch to amoxicillin-clavulanate. Initial multiplex PCR testing for common respiratory pathogens (influenza A and B, respiratory syncytial virus, human rhinovirus, adenovirus, Mycoplasma pneumoniae, and Chlamydia pneumoniae) was negative on admission and remained negative when repeated during disease progression.

Despite these interventions, the infant developed recurrent fever and paroxysmal spasmodic cough, which was accompanied by rising WBC (peaking at 37.60 × 10^9^/L; ANC 24.03 × 10^9^/L (63.9%) and lymphocytes 10.11 × 10^9^/L (26.9%)), PCT (9.062 ng/mL) and CRP (134.64 mg/L). Subsequently, pertussis polymerase chain reaction (PCR) was positive, confirming severe pertussis based on diagnostic criteria including lethargy, tachypnea (RR > 70 rpm), tachycardia (HR > 180 bpm), cyanosis, and the need for respiratory support [6,7]. Antibiotics were escalated successively to cefoperazone-sulbactam and piperacillin-tazobactam, with the addition of oral azithromycin. Elevated D-dimer (14.54 mg/L) prompted prophylactic anticoagulation with low-molecular-weight heparin.

**Figure 3:**
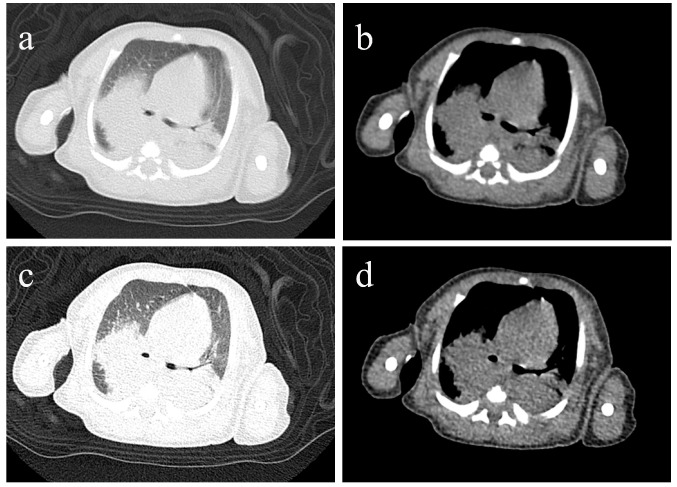
Chest CT scan of Case 1 prior to the first bronchoscopy (**a**–**d**). Images (**a**,**c**) are lung window settings, while images (**b**,**d**) are mediastinal window settings. They demonstrate increased bilateral lung markings, patchy opacities, and consolidation.

Persistent fever, tachycardia, and tachypnea were observed alongside a further rise in WBC to 52.25 × 10^9^/L (ANC 37.85 × 10^9^/L (72.4%) and lymphocytes 6.55 × 10^9^/L (12.5%)). Due to refractory hyperleukocytosis, two sessions of exchange transfusion (ET) were performed. Owing to ongoing consolidation (Figure 3), bronchoscopy with bronchoalveolar lavage (BAL) was undertaken. Targeted next-generation sequencing (tNGS) of the BAL fluid, which covers 220 common respiratory pathogens, identified macrolide-resistant *Bordetella pertussis* (MRBP) with the 23S rRNA A2047G mutation (640 reads, 65% frequency) and *Haemophilus* influenzae, with no other viruses, fungi, or atypical pathogens, effectively ruling out additional co-infections. After confirming normal glucose-6-phosphate dehydrogenase (G6PD) activity, hepatic and renal function, and obtaining informed consent for treatment, trimethoprim-sulfamethoxazole (TMP-SMZ) was administered at a dose of TMP 4 mg/kg plus SMZ 20 mg/kg twice daily. A second bronchoscopy was performed due to persistent consolidation on chest radiograph (Figure 2).

After 21 days of piperacillin-tazobactam and 14 days of TMP-SMZ treatment, follow-up laboratory tests showed a WBC of 9.49 × 10^9^/L (ANC 3.61 × 10^9^/L, 38.0%; lymphocytes 3.27 × 10^9^/L, 34.5%), PCT of 0.350 ng/mL, and CRP of 1.02 mg/L. The infant was subsequently discharged as cured after a 35-day hospitalization, with no echocardiographic evidence of PAH. Throughout the illness course, no clinical signs of pertussis encephalopathy (seizures, focal deficits, or altered mental status beyond sedation) were observed. Cardiac monitoring showed no arrhythmias or ST changes. Serial renal and hepatic function remained normal, with no acute injury despite transient hyponatremia.

### 2.2. Case 2

A 1-month-and-19-day-old male infant was admitted in spring 2025 with a 7-day history of cough that had exacerbated over the preceding 3 days. He was born full-term, the third child (G3P3), with all historical and perinatal details identical to Case 1 (including sick contact, maternal DTaP vaccination, delivery mode, perinatal conditions, maternal pregnancy, and parental health). Initially diagnosed with bronchitis, he received oral antitussives for one day without improvement. Subsequently, his cough became spasmodic and was accompanied by dyspnea, poor feeding, and dehydration, though he remained afebrile. After failing to improve following a 1-day course of ceftriaxone, dexamethasone, and N-acetylcysteine (NAC) at another hospital, he was transferred.

**Figure 4:**
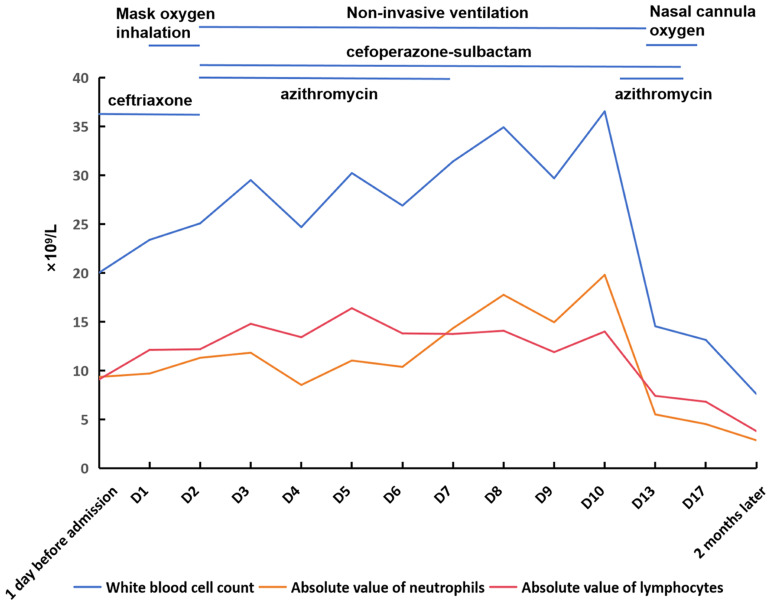
Changes in blood routine parameters and treatment of Case 2.

**Figure 5:**
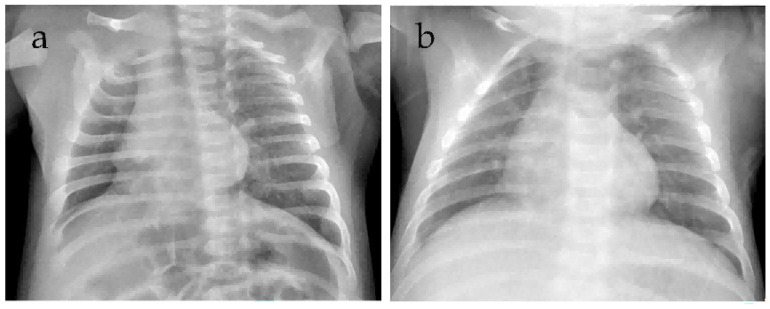
Chest radiograph images of Case 2 (**a**,**b**). Image (**a**) obtained at admission, demonstrating patchy opacity. Image (**b**) obtained 2 months after discharge, showing clear lung fields.

On admission, examination revealed cyanosis, lethargy, poor skin turgor, inspiratory retractions, and bilateral moist rales. Vital signs included HR 183 bpm, RR 72 rpm, and low peripheral oxygen saturation (SpO_2_) on room air. Laboratory investigations showed leukocytosis (WBC 20.02 × 10^9^/L, ANC 9.35 × 10^9^/L (46.7%), lymphocytes 9.09 × 10^9^/L (45.4%)), elevated PCT (0.636 ng/mL) and CRP (14.68 mg/L) (Figure 4), as well as hyponatremia. Chest radiograph demonstrated patchy opacity (Figure 5). He was diagnosed with severe pneumonia and moderate dehydration, and treatment was initiated with ceftriaxone, budesonide, fluid administration, and oxygen via mask.

Due to progressive hypoxemia, he was transferred to the intensive care unit (ICU) on day 2 and commenced on NIV. Concurrently, his WBC rose to 23.39 × 10^9^/L (ANC 9.70 × 10^9^/L (41.5%), lymphocytes 12.12 × 10^9^/L (51.8%)). Comprehensive multiplex PCR testing for common respiratory pathogens (same panel as in Case 1) was negative, and sputum culture showed no bacterial growth. Severe pertussis was suspected based on the presence of spasmodic cough and the need for respiratory support, and PCR testing confirmed *B. pertussis* infection [6,7]. Antibiotics were switched to cefoperazone-sulbactam combined with azithromycin; methylprednisolone was added to manage wheezing. His WBC peaked at 36.55 × 10^9^/L (ANC 19.80 × 10^9^/L (54.1%), lymphocytes 14.00 × 10^9^/L (38.3%)); but two echocardiograms revealed no evidence of PAH.

After two courses of azithromycin and 14 days of cefoperazone-sulbactam, his condition improved, enabling weaning from NIV to nasal oxygen. Given the favorable clinical response to empirical therapy, bronchoscopy with BAL and tNGS/mNGS was not pursued to further exclude co-infections. Throughout the illness course, findings regarding encephalopathy, cardiac monitoring, and organ function were identical to those described in Case 1. At discharge, the WBC was 13.14 × 10^9^/L (ANC 4.52 × 10^9^/L (34.5%), lymphocytes 6.81 × 10^9^/L (51.8%)), PCT was 0.413 ng/mL and CRP was 0.64 mg/L. A two-month follow-up demonstrated a normal WBC count of 7.6 × 10^9^/L (ANC 2.86 × 10^9^/L (37.7%), lymphocytes 3.80 × 10^9^/L (50.0%)), CRP of 0.24 mg/L and a clear chest radiograph (Figure 5).

## 3. Discussion

### 3.1. Rising Pertussis Incidence, Expanding Drug Resistance, and Compounding High-Altitude Risks

To provide context for this case series, we conducted a non-systematic literature review. We searched the following databases for articles published from database inception through January 2026: English databases: PubMed, Embase, Scopus, Web of Science, Cochrane Library, and Google Scholar. Chinese databases: CNKI (China National Knowledge Infrastructure), Wanfang Data, VIP (China Science and Technology Journal Database), and SinoMed (Chinese Biomedical Literature Database). Additional sources: Reference lists of relevant articles and medical history monographs were also screened for additional sources, with particular attention to historical literature on high-altitude flight therapy for pertussis from the 1930s–1950s.

Search terms were combined using Boolean operators as follows: Search terms: (“pertussis” OR “whooping cough”) AND (“high altitude” OR “hypobaric hypoxia” OR “altitude sickness”); (“pertussis” OR “whooping cough”) AND (“exchange transfusion” OR “leukoreduction” OR “hyperleukocytosis” OR “leukapheresis”); (“high altitude” OR “altitude”) AND (“therapy” OR “treatment”) AND (“historical” OR “flight” OR “chamber” OR “aviation”).

Global pertussis incidence has increased markedly, with 2024 WHO data showing a 13.8-fold increase globally and a 108.4-fold increase in China since 2020, respectively [8]. Infants younger than three months, who cannot yet complete vaccination or acquire maternal antibodies, bear the highest risk of severe disease [1]. Neonates are especially vulnerable. Nevertheless, data from high-altitude regions remain extremely limited. Concurrently, antimicrobial resistance in pertussis is expanding globally [3]. Although sporadic or modestly rising resistance has been reported in some settings [9], China has been experiencing sustained, large-scale outbreaks of MRBP, primarily due to 23S rRNA mutations [10,11]. In Shanghai, MRBP prevalence among children rose sharply from 36.4% (2016) to 98% (2024), predominantly driven by the MT28 ptxP3-MRBP clone [12,13]. National surveillance in 2024 reported 99.7% azithromycin resistance, with 99.5% of isolates belonging to the MT28-Shanghai clone and 48.0% remaining susceptible to TMP-SMZ [14]. This convergence of widespread resistance and high-altitude physiological challenges significantly complicates management of early-infant pertussis.

### 3.2. Pertussis Transmission Dynamics and Prevention in High-Altitude Settings

Health initiatives in China’s Xizang region have achieved historic progress, supported by national policies and assistance programs, with childhood vaccination coverage consistently >90% under the national immunization program [15,16]. Pertussis is generally well controlled in this region. Severe pertussis in young infants likely occurs primarily in those unvaccinated or with delayed vaccination resulting from illness or vaccine hesitancy; in vaccinated individuals, vaccine evasion by the MT28 ptxP3-MRBP clone may contribute [13]. However, waning immunity-protective antibodies decline within 3–5 years and are nearly undetectable by 12 years [17]-means adolescents and adults increasingly serve as asymptomatic reservoirs, driving pertussis transmission [3,18]. Both infants in this study had household contact with symptomatic individuals, consistent with evidence that family members—particularly siblings—are key sources [19,20]. In some high-altitude regions, such as parts of Ecuador, limited access to medical services may delay early health-seeking behavior [21]. Such geographical characteristics could thereby increase the risk of household transmission. To reduce pertussis transmission, the U.S. Centers for Disease Control and Prevention recommends routine tetanus, diphtheria, and acellular pertussis (Tdap) vaccination for all adolescents, with a single dose administered at 11–12 years of age. China is conducting clinical trials of Tdap vaccines for adolescents and adults. Concurrently, maternal Tdap vaccination provides passive protection in early infancy [1,3] and, although not yet routinely recommended in China, the 2025 national immunization program update now administers the first DTaP dose at 2 months of age and introduces a DTaP booster at 6 years.

### 3.3. Dual-Hit Pathogenesis of Severe Pertussis at High Altitude

Pertussis toxin (PT) induces hyperleukocytosis by inhibiting lymphocyte extravasation, resulting in intravascular leukocyte accumulation [22] and, subsequently, PAH through vascular infiltration and elevated blood viscosity [23]. The key immunological basis of severe pertussis is characterized by interleukin-8 (IL-8)-driven neutrophil hyperactivation, alongside IL-10-mediated immunosuppression and impaired Th1 responses [24]. PAH risk is significantly elevated at high altitude. The highly hypoxia-sensitive pulmonary vasculature in early infancy [7] triggers rapid vasoconstriction and pressure elevation, activates pro-inflammatory endothelial responses [25], and this endothelial activation may initiate pulmonary inflammation even without other pathogenic stimuli [26]. Combined with systemic hypoxia-driven inflammation (a “second hit”), these mechanisms promote hypoxic pulmonary hypertension (HPH) [25], increasing risk of right-heart failure or death in susceptible individuals [27]. High-altitude PAH pathogenesis further involves hyperviscosity and sympathetic activation, driving pulmonary vasoconstriction and vascular remodeling [28]. Sustained hypoxia during lung development promotes persistent vascular changes [29]; consequently, high-altitude infants often display right ventricular hypertrophy, impaired cardiopulmonary adaptation, and elevated baseline pulmonary vascular resistance [26]. Therefore, in high-altitude infants with pertussis, synergistic interaction between pre-existing pulmonary vascular dysfunction and pertussis-induced hyperleukocytosis substantially elevates the risk of cardiopulmonary deterioration.

### 3.4. Early Recognition and Aggressive Management in High-Altitude Settings

These findings underscore the necessity for enhanced monitoring and early identification of pertussis, especially at high altitude. In Case 1, timely leukoreduction (WBC 52.25 × 10^9^/L) likely prevented PAH, highlighting the importance of frequent WBC surveillance and proactive intervention. Early clinical recognition remains equally critical. Infants with paroxysmal cough, contact history, and hyperleukocytosis should undergo prompt PCR or metagenomic next-generation sequencing (mNGS) testing [30]. CRISPR (clustered regularly interspaced short palindromic repeats)/Cas (CRISPR-associated) technology also provides a highly sensitive alternative [31]. Both cases were PCR-confirmed. Case 1, with MRBP identified through tNGS, was effectively treated with timely administration of TMP-SMZ; Case 2, managed early based on clinical suspicion despite a lower WBC (23.39 × 10^9^/L), also achieved a favorable outcome. BAL offers additional diagnostic value in selected circumstances. While not first-line for initial pertussis confirmation due to its invasive nature, early BAL is invaluable when drug resistance is suspected or clinical response is poor. Given the rapid progression at high altitude, a lower threshold for early bronchoscopic evaluation in severe or refractory cases may facilitate timely resistance detection and optimize antimicrobial management. These experiences reinforce the need for frontline clinicians in high-altitude settings to pursue rapid diagnosis and timely, tailored management strategies.

### 3.5. Therapeutic Strategies for Drug-Resistant Pertussis in High-Altitude Infants

In vitro studies confirm sulfonamide efficacy against neonatal MRBP [32], but clinical reports remain limited to a 2006 French case involving a 4-month-old extremely preterm infant (corrected gestational age 43.3 weeks) [33] and two Japanese infants aged 1–2 months in 2025 [9]. Chinese multicenter data from 2024 indicated TMP-SMZ use in 55.5% of infants aged 0–2 months and in 59.6% of severe cases aged 0–4 years, though neonatal-specific data were lacking [14]. Potential neonatal risks—kernicterus, hepatorenal toxicity, bone marrow suppression—require careful evaluation. While 2024 Chinese guidelines contraindicated TMP-SMZ under 2 months of age [34], 2025 Chinese neonatal guidelines support conditional use [35]. Other susceptible agents include ceftazidime, cefoperazone-sulbactam, levofloxacin, and doxycycline [14]; piperacillin-tazobactam is recommended for severe cases under 2 months before transitioning to TMP-SMZ [36]. Based on our experience managing these two high-altitude infants, we offer the following observations. First, in high-altitude settings where the risk of PAH is elevated by environmental hypoxia, a cautious consideration of TMP-SMZ may be warranted even in young infants, provided that laboratory parameters (e.g., G6PD, hepatic and renal function) are normal and informed consent is obtained. Second, both cases underscore a critical gap in early management: although antibiotics were promptly switched upon suspicion of pertussis, confirmatory testing (PCR/mNGS) occurred only after ICU transfer. This delay highlights the need for earlier etiological suspicion and workup in general wards, particularly in resource-limited high-altitude areas where disease progression may be more rapid.

### 3.6. Exchange Transfusion in High-Altitude Pertussis: Lowering the Intervention Threshold

At high altitude, baseline leukocytes are higher, possibly reflecting an adaptive response to hypoxia [37]. Pertussis and high-altitude hypoxia can independently induce PAH [38,39]; their co-occurrence worsens prognosis. Early ET, performed before PAH onset, yielded greater benefit [40]. It removes circulating PT, reduces blood viscosity and leukocyte aggregation, and potentially halts PAH progression [2,23]. Timely ET prior to cardiopulmonary compromise can be lifesaving [7]. Wei et al. reported a 30-day-old infant (peak WBC 69.26 × 10^9^/L) who underwent double-volume ET; despite multiple complications, the infant survived, demonstrating that ET can stabilize cardiopulmonary status and “buys time” for other interventions [41]. Treatment stratified by WBC level (>50, >70, >100 × 10^9^/L) have been shown to reduce mortality [42]. Various ET thresholds have been proposed: WBC > 55 × 10^9^/L with tachypnea and/or tachycardia [43], or >40.0 × 10^9^/L [40]. The 2024 Chinese guidelines advise ET at WBC ≥ 50 × 10^9^/L, or ≥30 × 10^9^/L with a rising trend plus PAH or cardiopulmonary dysfunction, or with concurrent PAH or incipient cardiopulmonary failure [34]. The 2025 neonatal guidelines further specify the following criteria: (1) WBC > 25 × 10^9^/L with lymphocytosis (>12 × 10^9^/L) in the setting of shock, PAH, or organ failure; (2) peripheral WBC > 48 × 10^9^/L and lymphocyte count > 15 × 10^9^/L; (3) WBC > 30 × 10^9^/L and lymphocytes > 15 × 10^9^/L with a 50% increase within 24 h; or (4) other indicators such as persistent HR > 170 bpm, RR > 70 rpm, and SpO_2_ < 80% [35]. Notably, thresholds have progressively decreased over time. In our observation of Case 1, ET at WBC ≥ 50 × 10^9^/L pre-PAH likely contributed to recovery. Although not meeting all 2025 lymphocyte-based criteria, we propose that in high-altitude settings—with dual PAH risk from infection and hypoxic environment, as well as narrower compensatory reserve—ET timing should shift from “last resort” to “early intervention” at lower thresholds than at sea level.

### 3.7. Respiratory Management in High-Altitude Pertussis: Early Non-Invasive Ventilation

Early NIV helps prevent hypoxemia and secondary PAH. Infants under 3 months are at higher risk for requiring respiratory support, with independent risk factors including post-tussive cyanosis, fever, tachypnea, wheezing, corticosteroid use, and intravenous immunoglobulin administration [44]. In young infants, the chest wall is highly compliant due to cartilaginous ribs—approximately three times that of the lung in the neonatal period. This high compliance predisposes to chest wall deformation during increased respiratory effort, increasing work of breathing and reducing ventilatory efficiency, requiring activation of respiratory muscles to maintain chest wall stability. The neonatal diaphragm contains fewer fatigue-resistant type I muscle fibers, and infants are more dependent on diaphragmatic breathing, increasing their risk of respiratory fatigue [45]. During respiratory distress with increased inspiratory effort, paradoxical inward chest wall movement may occur, further compromising ventilation.

High-altitude infants exhibit additional adaptations to chronic hypoxia, including increased respiratory system compliance and enhanced ventilatory drive [46]. However, high chest wall compliance, when combined with reduced gas exchange surface area, leads to insufficient oxygen reserve relative to metabolic demand; once ventilation is compromised, oxygen saturation can decrease rapidly [47]. Both cases received prompt NIV upon ICU admission, likely contributing to the avoidance of significant hypoxemia and PAH. In the context of pertussis, spasmodic coughing imposes additional mechanical stress on the already highly compliant chest wall, exacerbating paradoxical movement and respiratory muscle load. Our experience suggests that early NIV is crucial not only for correcting hypoxemia but also for stabilizing the chest wall, reducing respiratory muscle workload, and improving ventilatory efficiency—particularly in high-altitude settings where NIV also delivers warmed and humidified oxygen, aiding airway hydration.

### 3.8. Ancillary Management: Anticoagulation Strategies and Supportive Therapies at High Altitude

High-altitude environments promote a hypercoagulable state through enhanced coagulation and impaired fibrinolysis [48], while pertussis-induced leukostasis contributes to hyperviscosity and microthrombosis [7,49], and hyperleukocytosis itself is associated with increased thrombotic risk [50,51]. Thus, infants with pertussis at high altitude face compounded thrombotic hazards. Monitoring D-dimer levels and considering prophylactic anticoagulation may help prevent overt disseminated intravascular coagulation (DIC) and reduce mortality. Additional markers—such as thrombin-antithrombin complex, thrombomodulin, α2-plasmin inhibitor-plasmin complex, and tissue plasminogen activator-plasminogen activator inhibitor complex—reflect activation of coagulation and fibrinolysis systems and may aid in early risk stratification [52]. In our series, Case 1 received early anticoagulation based on elevated D-dimer and tolerated it without complications; Case 2, with normal coagulation parameters, did not require anticoagulation. This experience suggests that judicious early anticoagulation may improve outcomes in severe pertussis at high altitude, even though neonatal evidence remains limited.

Furthermore, the characteristic thick respiratory secretions and inflammation-induced oxidative damage in pertussis represent important therapeutic targets. NAC possesses both mucolytic and antioxidant properties, with potential mechanisms including mucolytic action, antioxidant and anti-inflammatory effects, and synergistic antimicrobial activity [53]. A case report of severe pertussis demonstrated significant efficacy with repeated bronchoscopic NAC lavage, suggesting NAC may be incorporated into the management of mucus plugging [54]. At high altitude, hypoxia can exacerbate inflammatory injury, and animal studies have shown that NAC improves high-altitude hypoxia-induced acute lung injury by inhibiting oxidative stress [55]. Additionally, the dry, cold air at high altitude itself increases secretion viscosity, potentially offering compounded benefits from NAC in this setting. In Case 2, the infant had briefly received empirical therapy containing NAC before transfer; however, its independent effect is difficult to assess. But given the overlap between its multiple protective mechanisms and the pathogenic mechanisms of pertussis at high altitude, it holds potential therapeutic promise.

### 3.9. Historical Shift in Pertussis Treatment: Ascending, Descending, and Coexisting with Altitude

We reviewed historical literature on “pertussis and altitude” and identified an intriguing evolution in altitude-based management. During the 1930s–1950s, “high-altitude flight therapy” for pertussis was practiced in many countries, including Germany. Infants were flown to over 3000 m, and the whole process lasted 1.5 h, with reports of “marked improvement” in coughing [56]. Two 1991 BMJ letters later reflected on this practice [57,58]. Symptomatic relief may have related to distraction or altered breathing, but the mechanism was never validated, and significant risks prevented standardization. The therapy faded with antibiotics and vaccines. Over recent years, studies suggested that severe hypoxemia during respiratory infection increases infant mortality at high altitude [26]; conversely, descent to a lower elevation improves respiratory function in infants under one year with bronchiolitis [59]. For high-altitude pulmonary edema and cerebral edema, descent remains the cornerstone therapy [60,61]. For pertussis—with compounded risks of hyperleukocytosis and hypoxic PAH—this rationale is even stronger. In our clinical decision, we considered but did not pursue aerial transport because: (1) both infants were at a Lhasa tertiary center (3650 m) with ICU, NIV, and capability for ET and TMP-SMZ; (2) their critical illness made transport hazardous; and (3) the families expressed a preference not to transfer their children to other regions. Instead, we implemented aggressive on-site interventions at lower thresholds than at sea level—supported by recent healthcare improvements in Xizang [15,16].

To our knowledge, this is the first case series of severe pertussis in young infants residing at high altitude. The two cases presented here illustrate key differences from low-altitude reports. First, clinical progression is more rapid and severe: baseline hypoxemia, higher baseline leukocyte levels [37], and hypercoagulability [48] created a compromised host, with pertussis-induced hyperleukocytosis acting as a “second hit” on this reduced reserve, accelerating progression to critical illness and elevating PAH risk. Second, this risk profile necessitates more aggressive therapy: lower thresholds for NIV, prophylactic anticoagulation, and ET than in low-altitude settings. This experience illustrates a paradigm shift: from passive, risky altitude manipulation to active, pathophysiology-guided management at altitude. These findings identify high altitude as an independent risk modifier in severe infant pertussis, fundamentally altering disease presentation and management thresholds. This first clinical evidence from a high-altitude setting offers crucial insights for clinicians in similar environments globally. Proactive preventive measures should be reinforced to protect this vulnerable population. Nevertheless, descent remains relevant in resource-limited settings—for stable infants, transfer may be lifesaving; for the critically ill, on-site aggressive care offers the best chance.

## 4. Limitations

This study has several limitations. First, the inherent limitations of a two-case series apply. Second, follow-up duration differed between cases; however, both infants remained clinically stable post-discharge, as confirmed by telephone follow-up and pediatric health records. Imaging asymmetry (Case 2 had an incidental follow-up chest radiograph due to other illnesses; Case 1 did not minimize radiation) is a major limitation. Third, baseline differences exist (onset timing, resistance status), and Case 2 lacked BAL-mNGS to exclude co-infections—this should be considered when interpreting findings. Our aim is not to compare efficacy but to illustrate the clinical spectrum of severe pertussis in high-altitude infants. Standardized registries are needed.

## 5. Conclusions

In the context of China’s ongoing support initiatives in the Xizang region, the health status of this population has steadily improved. This first documented case series of pertussis in young infants from a high-altitude setting demonstrates that the disease follows a more severe clinical trajectory in this population, underscoring the need for comprehensive prevention, early recognition, and proactive management of serious complications—such as hyperleukocytosis, PAH, and thrombosis. Given the elevated risks of PAH and coagulation abnormalities at high altitude, we recommend actively evaluating leukoreduction interventions, such as ET, to mitigate the additional life-threatening risks imposed by high-altitude conditions.

## Figures and Tables

**Table 1:** Clinical data of two cases of pertussis infants. WBC: white blood cell; TMP-SMZ: trimethoprim-sulfamethoxazole; NIV: non-invasive ventilation; ET: exchange transfusion; *B. pertussis*: *Bordetella pertussis*; MRBP: macrolide-resistant *Bordetella pertussis*.

Case No.	Case 1	Case 2
Age	12-day-old	1-month-and-19-day-old
Gender	female	male
Gestational age	37 weeks and 5 days	full-term
Birth weight	2940 g	No data
Gravida and Para	G3P2	(G3P3)
Delivery mode	delivered vaginally	delivered vaginally
Pre-admission course	2-day	7-day
Parity	2	3
Peak WBC (×10^9^/L)	52.25	36.55
Antibiotic selection	cefoperazone-sulbactam, piperacillin-tazobactam, azithromycin, TMP-SMZ	cefoperazone-sulbactam, azithromycin
Respiratory support	NIV	NIV
Other special treatments	ET, anticoagulation	no
Pathogenic Results	MRBP	*B. pertussis*
Complications	respiratory failure, extreme hyperleukocytosis	respiratory failure, hyperleukocytosis
Outcome	Cured	Cured

## Data Availability

The original data supporting the findings of this study are available within the article. Additional inquiries regarding the data can be directed to the corresponding author.

## Abbreviations

The following abbreviations are used in this manuscript:

MRBP	macrolide-resistant *Bordetella pertussis*
*B. pertussis*	*Bordetella pertussis*
TMP-SMZ	trimethoprim-sulfamethoxazole
PAH	pulmonary arterial hypertension
DTaP	diphtheria, tetanus, acellular pertussis
HR	heart rate
bpm	beats per minute
RR	respiratory rate
rpm	breaths per minute
WBC	white blood cell
CRP	C-reactive protein
NIV	non-invasive ventilation
PCR	polymerase chain reaction
ET	exchange transfusion
BAL	bronchoalveolar lavage
G6PD	glucose-6-phosphate dehydrogenase
SpO_2_	peripheral oxygen saturation
ICU	intensive care unit
Tdap	tetanus, diphtheria, and acellular pertussis
DT	diphtheria-tetanus
PT	pertussis toxin
IL-8/IL-10	interleukin-8/interleukin-10
HPH	hypoxic pulmonary hypertension
t/mNGS	targeted/metagenomic next-generation sequencing
CRISPR/Cas	clustered regularly interspaced short palindromic repeats/CRISPR-associated
DIC	disseminated intravascular coagulation
ANC	absolute neutrophil count
